# Willingness to Use Gamified mHealth Apps Among Preadmission Patients With Pituitary Adenoma: Exploratory Sequential Mixed Methods Study

**DOI:** 10.2196/92924

**Published:** 2026-11-09

**Authors:** Xian Luo, Xiaoju Miao, Jing Shang, Lan Liu, Chaowei Hu, Xiaowen Wang, Zhongmin Fu

**Affiliations:** 1 Department of Nursing, Affiliated Hospital of Zunyi Medical University, No.149 Dalian Road, Huichuan District, Zunyi, Guizhou, 563000, China, 86 15120179663; 2 Department of Neurosurgery, Affiliated Hospital of Zunyi Medical University, Zunyi, Guizhou Province, 563000, China; 3 Department of Emergency, the First People's Hospital of Zunyi (The Third Affiliated Hospital of Zunyi Medical University), Zunyi, Guizhou, 563000, China

**Keywords:** pituitary adenoma, gamified mHealth apps, mobile health, fuzzy set qualitative comparative analysis, mixed methods research, serious games

## Abstract

**Background:**

Patients with pituitary adenoma (PA) must complete preparation tasks during the preadmission stage and may experience information asymmetry and anxiety. Gamified mobile health (mHealth) apps may support motivation and engagement; however, research has examined isolated predictors, and the combinations of app-related factors associated with willingness to use these tools remain unclear.

**Objective:**

This study aimed to identify factors associated with willingness to use gamified mHealth apps among preadmission patients with PA and to examine configurations associated with high and low willingness.

**Methods:**

We conducted an exploratory sequential mixed methods study comprising a qualitative phase followed by a cross-sectional quantitative phase, guided by the stimulus-organism-response framework. Participants were recruited using criterion-based purposive sampling from the Department of Neurosurgery at the Affiliated Hospital of Zunyi Medical University between August and November 2024. In the qualitative phase, 10 patients completed semistructured interviews after viewing a standardized description and slide presentation of a hypothetical gamified mHealth app. Interview data were analyzed through inductive open coding followed by deductive framework-guided categorization. The findings informed a 27-item checklist covering 6 antecedent conditions and willingness to use, refined through multidisciplinary expert consultation. In the quantitative phase, 120 patients completed a cross-sectional survey. Variables were directly calibrated as fuzzy sets, and fuzzy-set qualitative comparative analysis was used to assess necessity and identify configurations associated with high and low willingness.

**Results:**

The qualitative analysis identified reward mechanisms, interactivity, enjoyment, privacy protection, usability, and authority background as app-related conditions. No single condition met the prespecified necessity threshold. Four configurations were associated with high willingness: reward-driven engagement (H1), interactive enjoyment with privacy protection (H2), privacy- and usability-supported enjoyment (H3), and authority-supported functional assurance (H4). Configuration consistency ranged from 0.960 to 0.997; overall solution consistency and coverage were 0.964 and 0.510, respectively. Five configurations were associated with low willingness: broad feature deficit (L1), authority with engagement and usability deficits (L2), enjoyment and privacy deficit (L3), enjoyment and privacy deficit despite reward and authority support (L4), and authority-background deficit (L5). Their consistency ranged from 0.994 to 1.000; overall solution consistency and coverage were 0.993 and 0.645, respectively.

**Conclusions:**

By integrating patients’ qualitative accounts with configurational analysis, this study extends single-factor research and provides disease- and stage-specific evidence for preadmission care in PA. The configurations offer a preliminary basis for modular prototypes: rewards, progress indicators, and interactive checklists could support preparation tasks; interactive education could be combined with visible privacy controls; simplified navigation could improve access to examination and hospitalization instructions; and hospital endorsement and clinician-reviewed content could strengthen information credibility. Because participants evaluated a hypothetical rather than functioning app, these findings concern stated willingness, not actual adoption or clinical effectiveness. Prospective studies using deployed apps, objective task-completion measures, and app-log data are required.

## Introduction

Pituitary adenoma (PA) is a prevalent primary brain tumor in adults. Recent epidemiological data indicate an age-standardized incidence of up to 10.1 out of 100,000 and an overall prevalence of approximately 175.1 out of 100,000, highlighting that PA is not uncommon and may be increasing in frequency [1]. Transsphenoidal surgery has been the primary surgical treatment, with most patients undergoing elective procedures. In China, most hospitals require patients to undergo a preoperative at-home waiting period of several days to weeks prior to formal admission. Rather than a passive interval, this stage represents a critical window in which patients must complete key preparatory tasks, including acquiring disease-related knowledge, arranging preoperative examinations, adjusting medications and lifestyle behaviors, and undertaking psychological preparation. However, without timely support from health care professionals, patients frequently experience psychological distress and unmet information needs [2,3]. On the one hand, a tumor diagnosis is frequently accompanied by anxiety, depression, and feelings of uncertainty, and some patients also experience symptoms such as visual impairment, headache, and cognitive dysfunction. On the other hand, preoperative health management relies heavily on patients’ self-management, yet the lack of continuous, authoritative, and easily comprehensible professional support often results in inadequate preparation and reduced adherence. Among patients undergoing elective surgery, adherence to preoperative instructions remains suboptimal. A quality-improvement study in a preanesthesia assessment clinic reported incomplete adherence rates of up to 38.1% for perioperative medication instructions and 8.8% for fasting instructions, suggesting that conventional preoperative management approaches—largely dependent on verbal or paper-based education—may be insufficient to ensure the quality of out-of-hospital preparation [4].

Mobile health (mHealth) apps, owing to their convenience and capacity for functional integration, have been widely adopted in preoperative management [5,6]. Although patients often express initial interest in mHealth, real-world use is frequently characterized by low engagement after download or rapid attrition, commonly attributed to monotonous content, complex interfaces, limited personalized feedback, and concerns regarding trust and privacy [7,8]. In a single-center retrospective cohort of 549 patients undergoing elective surgery within a colorectal surgical service, 473 (86.2%) activated a digital patient-engagement platform. Among those who activated the platform, 382 (80.8%) completed at least 1 patient-reported outcome survey, 259 (54.8%) completed at least 1 in-hospital health check, and 270 (57.1%) completed at least 1 postdischarge health check during the perioperative follow-up period [9]. These differences between platform activation and subsequent task completion indicate that initial adoption alone may not translate into sustained engagement with digital health interventions. Gamified mHealth apps incorporate game elements into mobile platforms to enhance users’ motivation to engage in health-related activities, thereby promoting sustained healthy behaviors [10]. Seaborn and Fels [11] defined gamification as the integration of game elements, mechanics, features, design, and structure within nongame contexts to achieve nongame objectives. By introducing game elements such as points, badges, leaderboards, and virtual rewards, gamified mHealth apps address the drawbacks of traditional mHealth apps, such as monotonous content, complex operations, and lack of personalization, to enhance user engagement and motivation. This approach has shown promising potential in improving technology acceptance [12,13].

However, existing research on the acceptability of gamified mHealth apps has largely focused on contexts such as long-term chronic disease management, health promotion, or symptom follow-up, with the primary goal of enhancing sustained adherence and engagement over time [14,15]. By contrast, evidence remains limited for short-cycle, task-intensive health care transition contexts characterized by substantial uncertainty. For patients with PA, a clearly defined time window requires the completion of multiple key preadmission tasks outside the hospital while simultaneously coping with the psychological burden associated with diagnosis and surgical decision-making. This setting differs from chronic disease follow-up or long-term symptom management in its temporal structure, task density, and modes of professional support.

Moreover, many prior studies have relied on conventional statistical approaches that emphasize the linear effects of independent variables on behavioral intention, while overlooking potential interactions among determinants [16]. Therefore, this study adopts a mixed methods design to systematically examine PA preadmission patients’ intentions to use gamified mHealth apps and the underlying mechanisms. Qualitative interviews were used to generate candidate antecedent factors and clarify key concepts, whereas explanatory pathways were subsequently identified using qualitative comparative analysis (QCA). QCA offers a novel methodological lens for understanding multicondition configurations and pathway diversity in behavioral decision-making [17]. By emphasizing equifinality and causal asymmetry, QCA facilitates the identification of how different combinations of factors jointly constitute configurations associated with high use intention.

The stimulus-organism-response (SOR) model was originally proposed by Mehrabian and Russell [18] to explain how external environmental stimuli influence behavioral responses through individuals’ cognitive and affective states, emphasizing that behavioral outcomes are not directly triggered by a single factor but are mediated by internal psychological processing. In recent years, the SOR model has been widely applied in research on online user behavior [19,20]. In the context of mHealth, an app’s functional design, interaction modalities, and modes of information presentation can be conceptualized as stimuli; patients’ cognitive appraisals, affective responses, and perceived trust constitute the organism state; and intention to use and sustained engagement represent the response [21]. During the preadmission period, patients with PA face multiple concurrent stressors, including disease-related uncertainty, intensive preoperative task demands, and limited real-time professional support. As a result, technology adoption behaviors in this setting are often shaped jointly by emotional experience, trust judgments, and cognitive load. The SOR model is therefore well suited to elucidating an integrated emotion-cognition-behavior decision-making mechanism in high-risk health care contexts. Guided by SOR theory, this study conceptualizes the design features of gamified mHealth apps (eg, reward mechanisms, interactivity, enjoyment, privacy protection, usability, and perceived authority) as external stimuli; patients’ emotional responses and cognitive evaluations as organism states; and use intention as the behavioral response outcome. This framework provides a clear theoretical basis for developing the interview guide, screening antecedent factors, and conducting subsequent QCA configurational analyses.

## Methods

### Study Design

This study used an exploratory sequential mixed methods design comprising a qualitative interview phase followed by a cross-sectional quantitative survey phase. This design uses a qualitative-first, quantitative-second sequence to explore a phenomenon and subsequently examine the qualitative findings in a quantitative sample [22]. The qualitative phase was conducted to identify relevant conditions, whereas the quantitative phase was used for configurational pathway analysis. First, semistructured interviews were performed to preliminarily explore potential determinants of preadmission patients’ intention to use gamified mHealth apps. Next, guided by the SOR framework, the emergent themes were conceptually categorized and mapped to their theoretical positions at the stimulus (S), organism (O), or response (R) level. Based on these results, an initial pool of questionnaire items was developed with reference to established scales, and wording was refined through expert consultation to ensure content validity. The finalized questionnaire was subsequently administered to the quantitative sample, and its reliability and construct validity were evaluated using the survey data. The survey data were then analyzed using fuzzy-set qualitative comparative analysis (fsQCA) to identify configurations associated with willingness to use gamified mHealth apps. The operationalization of the SOR framework across the study phases is presented in Figure 1.

**Figure 1.**
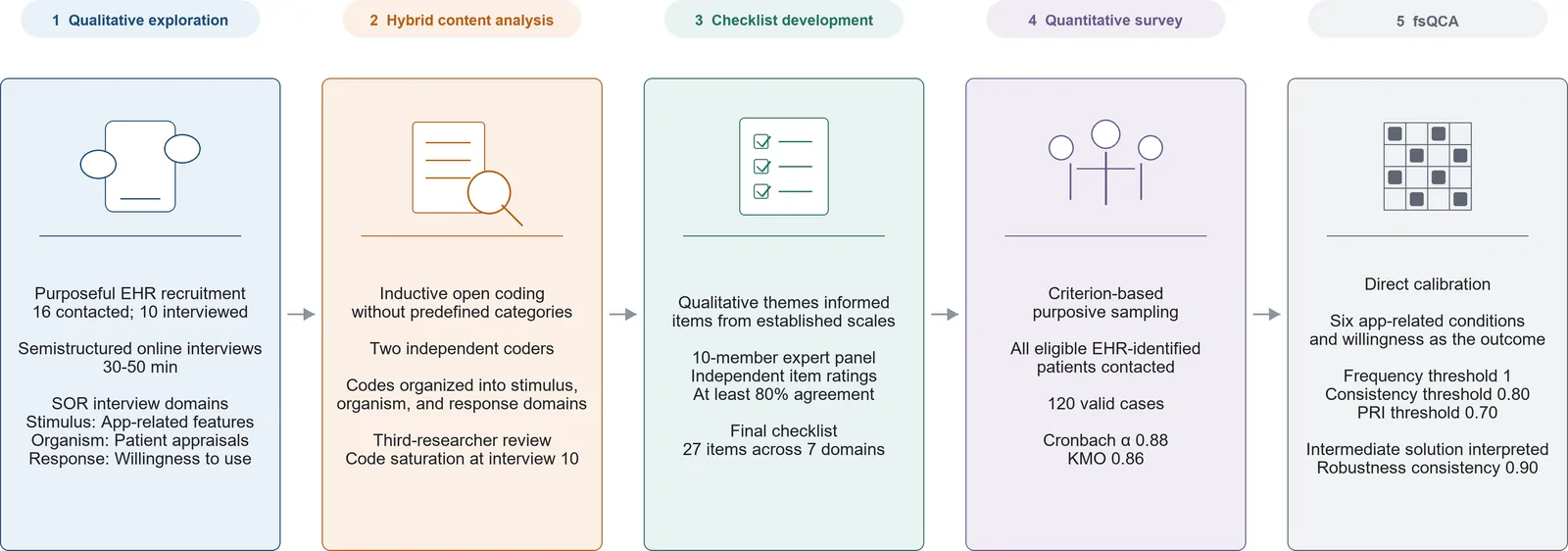
Stimulus-organism-response (SOR)–guided exploratory sequential mixed methods study design. EHR: electronic health record; fsQCA: fuzzy-set qualitative comparative analysis; KMO: Kaiser-Meyer-Olkin measure of sampling adequacy; PRI: proportional reduction in inconsistency.

### Ethical Considerations

The study was approved by the Ethics Committee of Zunyi Medical University (KLL-2024‐024) and registered with the Chinese Clinical Trial Registry (ChiCTR2500096761). Before participation, interview participants provided electronic informed consent, including consent for audio recording, and survey participants provided written informed consent. Interview transcripts and survey datasets were deidentified and labeled using study codes rather than names or other direct identifiers. Potentially identifying information was removed from reported quotations. Electronic files, including audio recordings, transcripts, and survey data, were stored on password-protected computers and were accessible only to authorized members of the research team. All included patients filed informed consent for data use. The results were reported in aggregate, except for deidentified interview quotations. Participants in both the qualitative and quantitative phases received no monetary or nonmonetary compensation for participation. Neither the manuscript nor the supplementary materials contain photographs or other images from which individual participants could be identified.

### Qualitative Research Phase

#### Theoretical Framework

SOR theory [23] was used as the theoretical framework to systematically explain the factors influencing willingness to use gamified mHealth apps. According to SOR theory, environmental stimuli affect an individual’s cognitive, emotional, and physiological states, which subsequently influence their behavioral responses [18]. The SOR theory has been widely applied in online user behavior studies [19,20] and provides a concise and structured way to explore the factors affecting willingness to use gamified mHealth apps.

#### Participants

Purposeful sampling was used to recruit patients with PA who were scheduled for hospitalization in August 2024 and were identified through the electronic health record (EHR) system. The inclusion criteria were as follows: (1) aged 18 years or older, (2) had completed at least middle school education, (3) had a confirmed diagnosis of PA, (4) owned and were able to use an Android or iOS smartphone, (5) had used an mHealth app within the previous month, (6) were able to understand the interview questions and communicate their experiences, and (7) voluntarily agreed to participate and provided informed consent. The exclusion criteria were severe sensory impairment, cognitive impairment that prevented completion of the interview, and a history of psychiatric disorders.

Potential participants received a standardized description of the study before deciding whether to participate. A total of 16 patients were contacted by telephone. Two were excluded because severe visual impairment prevented the completion of the interview, 3 did not meet the smartphone-use eligibility criterion, and 1 declined to participate. The remaining 10 patients completed the semistructured interviews. Data collection and preliminary analysis were conducted concurrently, and recruitment ended after the research team determined that no additional themes relevant to the research question were emerging from the interviews.

#### Data Collection

Since prehospitalization patients with PA had not been admitted to the hospital, face-to-face interviews were not feasible. Therefore, semistructured interviews were conducted via Tencent Meeting, with only the researcher and participant present in the virtual room. The interview outline (Multimedia Appendix 1), developed based on relevant literature and expert opinions, focused on factors influencing the willingness of patients with PA to use gamified mHealth apps. The first part of the interview explored three dimensions: (1) stimulus (eg, game features, information presentation formats), (2) organism (eg, patients’ cognitive evaluation, emotional states), and (3) response (eg, willingness to use, inclination toward continued use). The second part introduced existing gamified mHealth apps and personalized features through a slide presentation (Multimedia Appendix 2), inviting participants to share their opinions on the design and functionality. Each interview lasted between 30 and 50 minutes. Interviews were conducted by a researcher who held a PhD and had specialized training in qualitative research. Before the interview, participants completed a brief questionnaire to gather demographic data (age, ethnicity, education level, marital status) and smartphone usage information (Multimedia Appendix 1). All interviews were conducted in Chinese, recorded in real-time, and transcribed verbatim within 48 hours. Transcripts were sent to participants for feedback to ensure accuracy. All data were anonymized and securely stored on password-protected computers.

Data collection and preliminary coding were conducted concurrently. After each interview, 2 researchers independently reviewed the transcript and updated the cumulative codebook. Code saturation was defined as the point at which consecutive interviews generated no new codes or categories relevant to the research question. Interviews 9 and 10 elaborated existing categories but generated no new codes or categories; recruitment therefore ended after the 10th interview. This procedure was consistent with evidence that code saturation may be reached after approximately 9 interviews in studies with a focused research aim [24].

#### Data Analysis

The interview data were analyzed using a hybrid inductive-deductive content analysis approach [25]. In the first stage, 2 trained researchers conducted open coding without predefined categories, allowing the initial codes to emerge inductively from the data while retaining participants’ original experiences and expressions. In the second stage, the researchers reviewed and integrated the initial codes and deductively organized them according to the stimulus, organism, and response domains of the SOR framework. NVivo (version 14.0; Lumivero) was used to facilitate data management and coding. A third researcher reviewed the preliminary coding framework and findings. Discrepancies were discussed by the research team until consensus was reached. The final themes were supported by representative interview excerpts.

### Quantitative Research Phase

#### Participants

The quantitative phase was a cross-sectional study conducted from September to November 2024 at the Department of Neurosurgery, Zunyi Medical University Hospital. Criterion-based purposive sampling was used. EHRs were reviewed to identify patients with PA scheduled for admission. All patients who met the eligibility criteria during the study period were contacted and invited to participate; no further selection was made among eligible patients. A total of 120 patients participated in the survey assessing willingness to use gamified mHealth apps. The inclusion and exclusion criteria were identical to those used in the qualitative phase. Participants completed the questionnaire during hospitalization registration, which took approximately 10 minutes.

#### Design and Development of the Quantitative Survey Instrument

The research tools included a general information survey and a screening checklist for factors influencing the willingness of prehospitalization patients with PA to use gamified mHealth apps. The general information survey was designed by the researchers and included demographic details such as age, gender, education level, occupation, familiarity with smart devices, frequency of smart device usage, and health information sources.

To develop a screening checklist of determinants of intention to use gamified mHealth apps among preadmission patients, we first extracted factors repeatedly emphasized in the qualitative data, including patients’ cognitive appraisals, emotional responses, and behavioral intentions toward gamified mHealth apps. Next, the identified themes were mapped onto the SOR framework to clarify their theoretical attributes. Specifically, themes describing app features (eg, reward mechanisms, interactivity, fun, privacy protection, authority background, and usability) were classified as stimulus-level factors, whereas themes reflecting patients’ psychological states (eg, trust, positive affect, and social identification) were categorized as organism-level constructs; willingness to use the app was defined as the response-level outcome. These constructs informed the theoretical development of the item pool; however, the quantitative checklist operationalized the 6 app-related condition domains and willingness to use as the outcome domain. Finally, an initial item pool for the checklist was generated by drawing on established measurement scales, and the items were further refined through expert consultation to ensure content relevance, clarity of wording, and conceptual consistency. The panel comprised 3 neurosurgeons, 3 specialist nurses, 2 software engineers, and 2 patient representatives. Panel members independently evaluated each item for relevance, wording clarity, and importance. For items with discrepant ratings, retention was determined using an 80% agreement criterion; an item was retained when at least 80% (8/10) of the panel members rated it as important. Item wording was revised according to the experts’ written feedback. The finalized checklist is provided in Multimedia Appendix 3. The final version comprised 27 items across 7 domains: reward mechanisms, interactivity, fun, privacy protection, ease of use, authoritative background, and willingness to use. Users rated their agreement on a 5-point Likert scale, ranging from “strongly disagree” (1) to “strongly agree” (5). The questionnaire demonstrated good internal consistency (Cronbach *α*=0.88). The KMO (Kaiser-Meyer-Olkin) measure of sampling adequacy was 0.86, and the Bartlett test of sphericity was significant. All participants completed the survey at the time of their hospitalization registration.

#### Sample Size Estimation

No universally accepted power-based formula is currently available for determining the minimum sample size required for fsQCA [26,27]. Methodological literature indicates that fsQCA is applicable to small- and medium-sized samples and that a case-frequency threshold of 1 may be used in such studies, whereas a higher threshold is generally recommended for large samples, such as those comprising 150 or more cases [28]. Previous empirical fsQCA studies have also used a frequency threshold of 1 with comparable sample sizes, including studies involving 108 [29] and 117 cases [30]. Accordingly, all eligible and consenting preadmission patients identified through the EHRs during the predefined recruitment period were included in the present study, resulting in 120 valid cases. This sample was considered adequate for the exploratory, case-oriented configurational analysis, and the case-frequency threshold was set at 1 to retain empirically observed configurations.

#### Data Analysis

Categorical data were presented as percentages, and continuous data were presented as means (SD). *χ*^2^ tests and 2-tailed independent-samples *t* tests were used for baseline comparisons. To explore the pathways influencing willingness to use gamified mHealth apps, fsQCA was used. This method, widely used in management studies, investigates the joint effects of multiple antecedents [17]. The analysis proceeded in four steps [31,32]:

1. Data calibration: Since all conditions and outcome variables in this study were derived from a 5-point Likert scale, the “direct calibration” method was used. The observed mean was defined as the crossover point of the fuzzy set, with the maximum value minus 1 set as the full membership threshold and the minimum value set as the full nonmembership threshold.
2. Necessity testing: Before conducting the configuration analysis, each condition’s necessity was tested individually. Consistency and coverage scores were calculated to determine the necessity and sufficiency relationships between variables. A condition was considered necessary if its consistency score exceeded 0.9.
3. Configurational sufficiency analysis: A truth table was constructed using a consistency threshold of 0.80, a frequency threshold of 1, and a proportional reduction in inconsistency threshold of 0.70. The standard analysis generated 3 types of solutions: complex, parsimonious, and intermediate solutions. The intermediate solution was used as the primary basis for interpretation because it incorporates theoretically plausible directional expectations while allowing the identification of core and peripheral conditions through comparison with the parsimonious solution.
4. Robustness testing: To test the robustness of the configurations, local parameters were adjusted. The consistency threshold was raised from 0.80 to 0.90 while keeping other settings constant. If the configuration results remained unchanged, it indicated that the configuration was robust. The quantitative analysis was conducted using IBM SPSS Statistics (version 19.0) and fsQCA software (Ragin and Davey).

## Results

### Participant Flow

Participant recruitment, eligibility screening, exclusions, and final inclusion in the qualitative and quantitative phases are shown in Figure 2.

**Figure 2.**
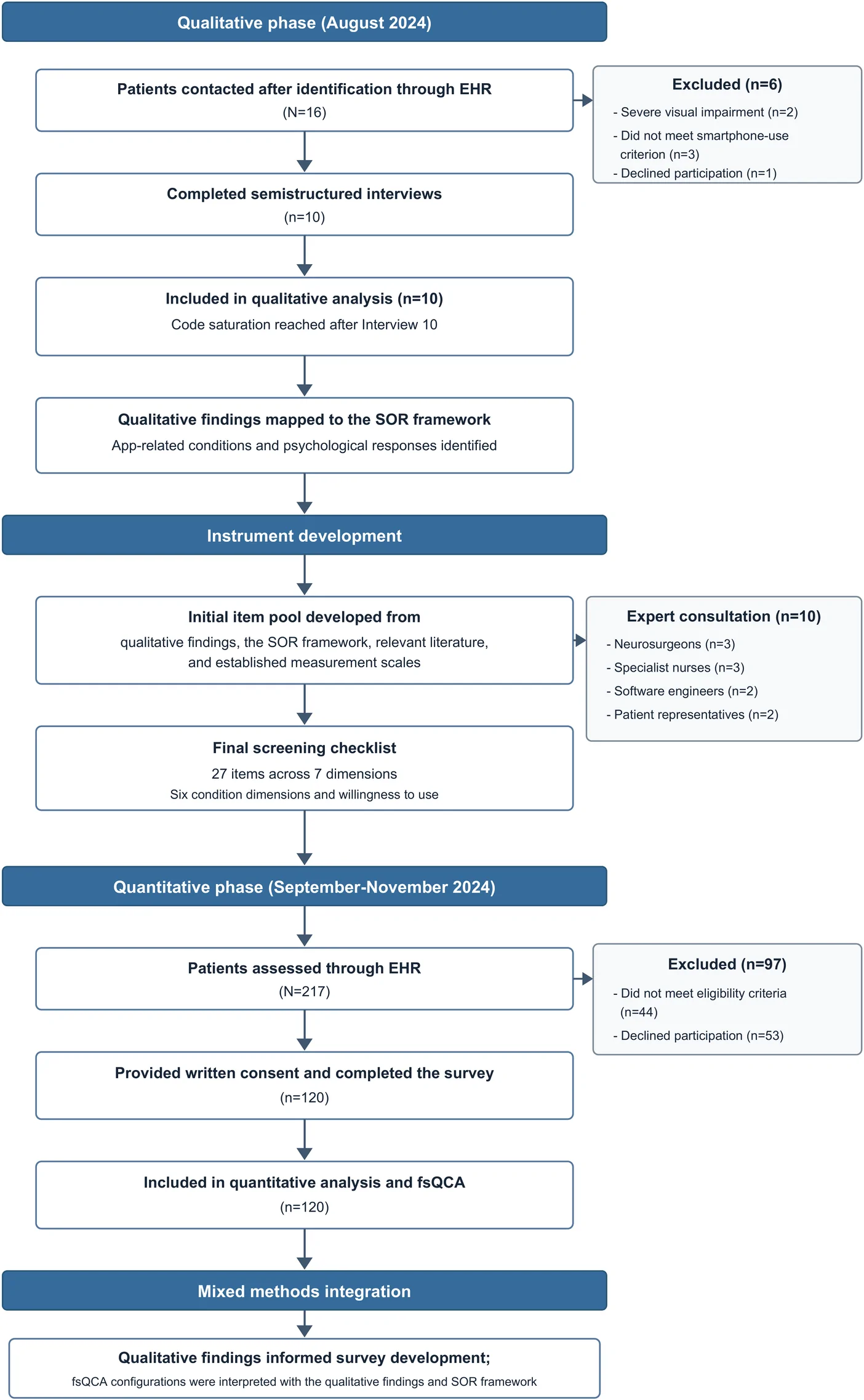
Study procedures and participant flow through the exploratory sequential mixed methods study. EHR: electronic health records; fsQCA: fuzzy-set qualitative comparative analysis; SOR: stimulus-organism-response.

### Qualitative Results

#### Respondent Demographics

Detailed information about the respondents is shown in Table 1. Among the 10 respondents, the average age was 38.80 (SD 15.01) years. All participants owned smartphones, and most patients expressed interest in gamified mHealth programs (n=9), though only a few were familiar with mobile gaming (n=4).

**Table 1.** Characteristics of qualitative study participants.

Variables	Values
Sex, n (%)	
Male	5 (50)
Female	5 (50)
Age (y), mean (SD)	38.80 (15.01)
Marital status, n (%)	
Married	8 (80)
Unmarried	2 (20)
Education level, n (%)	
Middle school	4 (40)
High school/technical school	4 (40)
College/university	2 (20)
Disease type, n (%)	
Functional PA^a^	8 (80)
Nonfunctional PA	2 (20)
Symptoms, n (%)	
Headache	6 (60)
Hormonal imbalance	4 (40)
Smartphone ownership, yes, n (%)	10 (100)
Use smartphone daily, yes, n (%)	10 (100)
Used health app to learn about disease, yes, n (%)	4 (40)
Interest in gamified mHealth apps, yes, n (%)	9 (90)
Familiarity with mobile games, yes, n (%)	4 (40)

a PA: pituitary adenoma.

#### Interview Results

Using the SOR theory, 2 main themes were identified from the interview data: stimulus factors influencing willingness to use gamified mHealth programs and organism responses to these stimuli. Specific themes and illustrative quotes are summarized in Table 2.

**Table 2.** Interview results: themes and illustrative quotes.

Theme and subtheme	Illustrative quote
Stimulus factors	
Reward mechanism	“It feels like getting MVP^a^ in a game;...if points could be exchanged for priority questions with the lead surgeon, it’d be far more exciting than reading dry articles!” (P02) “I think if, like the domestic sports app KEEP, completing daily health tasks could earn ‘points’ that could be exchanged for real rewards,...I’d be more motivated to keep using it.” (P08)
Interactivity	“Many apps feel cold like machines,...if I could interact with other patients, or even discuss and share experiences, I’d feel more accomplished.” (P01) “I don’t like direct communication with others, I prefer to do my own thing in peace, but virtual interaction in games, even if it’s virtual, I still feel connected to my treatment.” (P03)
Fun	“I think this gamified method is interesting,...it feels more like playing a game....If I had to just listen to instructions every day, I’d probably get bored.” (P09) “Combining games and healthcare, I’m still a bit skeptical, but thinking about it,...it’s fun,...fun makes it easier to turn this into a daily habit rather than a task I must complete.” (P04)
Privacy protection	“I don’t like sharing my condition with others,...when I was in the hospital and the nurse called out my name loudly, someone recognized me and asked, which made me uncomfortable.” (P07) “Cancer is something anyone can get, but most people don’t understand my condition. When they hear ‘tumor,’ they start asking a lot of questions,...I don’t want people to know.” (P05)
Ease of use	“At my age, I’m not interested in apps that are flashy or complicated. If it requires too much effort to figure out, I won’t bother....I want a hassle-free solution for my health.” (P06)
Authoritative background	“There are so many fitness and recovery programs online, but I don’t trust them....If it comes from a reputable hospital, I’d feel much more comfortable using it.” (P09)
Organism response	
Positive emotional response	“I like the interesting design, it makes me feel happy, especially during illness.” (P04) “It’s like having a friend who gives me reminders, telling me what to do at each stage, and even keeps in touch with the doctor. This reassurance is very comforting.” (P01)
Positive cognitive response	“I feel this program is one I could use....Don’t make me spend time figuring out how it works, it’s best if I can understand it at a glance.” (P06) “It should be as simple as turning on a light switch, just a click. If it’s as easy as chatting on WeChat, I’d be willing to try it.” (P09)
Low risk perception	“I’ve heard too much about data leaks. When I first heard about the program, I was worried. But you said the program encrypts personal information, only myself and the doctor can see it, which made me feel much safer.” (P07) “If I have to enter my condition and health data, I’ll need to ask where the data is stored, who can see it, and if it might leak. But if it’s from a reputable hospital and certified for security, I’d feel more comfortable.” (P10)
Social recognition	“Since I got sick, I’ve been isolated at home without much interaction with others....If I could compare with others and see who’s doing better, it would motivate me.” (P02) “The reward system is nice. If I can get a badge after completing tasks, or a ‘Star Performer’ title, it’ll help me feel progress, and others will see my efforts.” (P10)

a MVP: most valuable player.

In the stimulus factors theme, reward mechanisms were seen as an explicit behavior driver, evoking not only goal-oriented responses but also a “gamified self-discipline” mechanism. Many respondents compared this to “playing games” or “earning rewards.” This symbolic reward was internalized as a “sense of achievement” and a symbol of “rehabilitation motivation.” In the interactivity dimension, respondents exhibited a contradictory mindset: they desired social support and empathy from peers while being cautious about the emotional burden of traditional social interactions. Virtual interaction, with its non-face-to-face nature, became a “social compromise for emotional safety.” Emphasis on fun showed that patients resist the “monotony” of managing their illness daily, and the gamification made the recovery process feel “lighter” and “more integrated into daily life.” Privacy protection was often highlighted in interviews, especially in contexts involving disease labeling and social scrutiny. The program’s anonymity was crucial for maintaining patients’ dignity and boundaries.

In the “organism response” theme, participants frequently mentioned positive emotional reactions, such as feeling “accompanied,” “gently reminded,” or like “having a friend,” showing that health programs were seen not just as tools but as “emotional agents.” In the positive cognitive response dimension, participants valued program features such as “authoritative sources,” “simple operations,” and “efficient reminders,” reflecting a psychological need for control and predictability in the gamified mHealth program. This indicated a trust crisis and a compensatory mechanism in the face of modern medical uncertainties. Meanwhile, questions about low-risk perceptions (eg, information security and data use) showed that patients with chronic diseases were especially vigilant in the information age, making behavioral choices within a “risk-protection” cognitive framework. Finally, social recognition, a common underlying logic, reflected a shift from isolated recovery to a “visible” role within a collective comparison and sharing context. By displaying “visual achievements” and “behavioral traces,” patients aimed to rebuild self-efficacy and group belonging in the digital space.

Based on the interview results, we developed an SOR theory framework to illustrate the factors influencing the willingness of prehospitalization patients with PA to use gamified mHealth apps (Figure 3).

**Figure 3.**
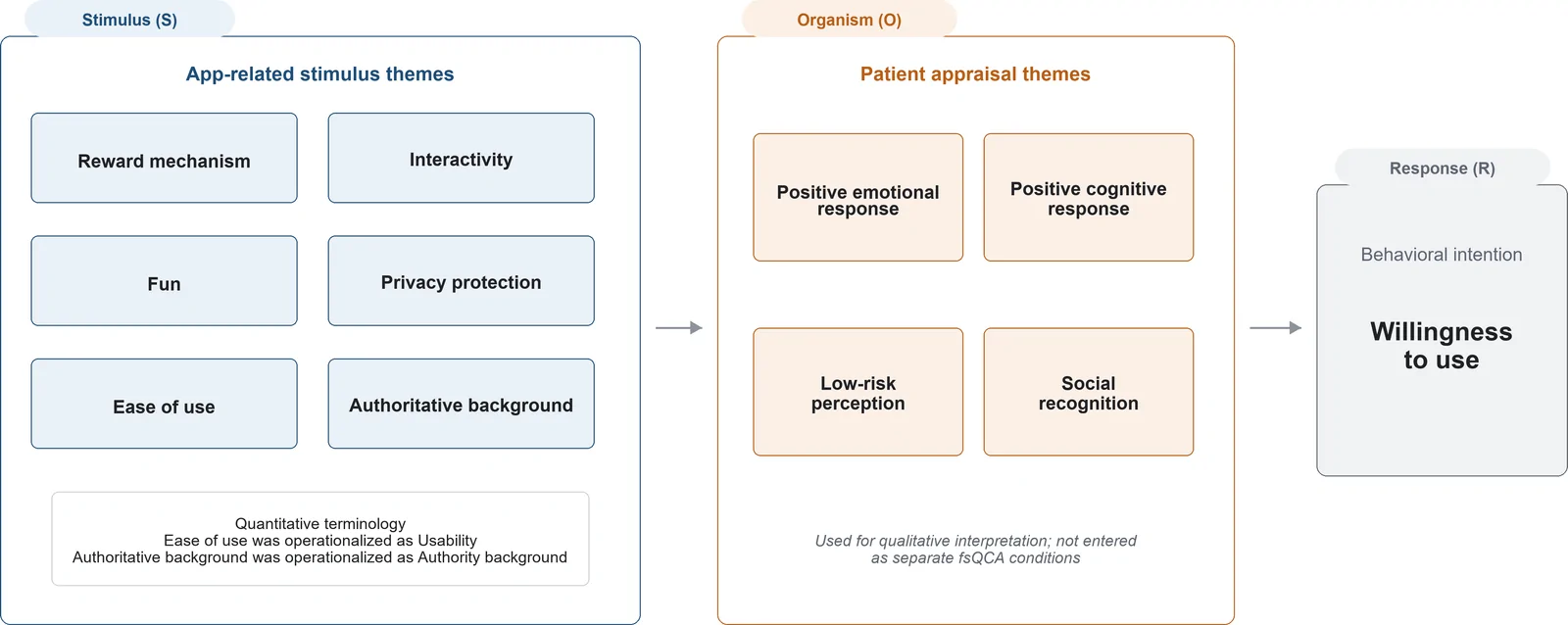
Qualitative thematic map within the stimulus–organism–response framework (SOR)*.* Six app-related stimulus themes and 4 patient-appraisal themes were organized within the SOR framework in relation to willingness to use gamified mHealth apps. Organism-level themes informed qualitative interpretation but were not entered as separate conditions in the fuzzy-set qualitative comparative analysis (fsQCA).

### fsQCA Analysis Results

#### Variable Calibration

The scores obtained from the screening checklist were used as the condition variables, and willingness to use gamified mHealth apps was used as the outcome. All condition and outcome variables were calibrated using the direct calibration method. For each variable, the observed maximum score minus 1 was specified as the threshold for full membership, the observed mean as the crossover point, and the observed minimum score as the threshold for full nonmembership. The calibration anchor points for each variable are detailed in Table 3.

**Table 3.** Descriptive statistics and direct calibration anchors for the conditions and outcome among pre-admission patients with pituitary adenoma (N=120)^a^.

Variable type	Variable	Full membership	Crossover point	Full nonmember ship	Mean (SD; range)
Condition	Reward mechanism	3.75	3.08	1.00	3.08 (1.35; 4.75-1.00)
Condition	Interactivity	4.00	3.31	1.00	3.31 (1.09; 5.00-1.00)
Condition	Enjoyment	4.00	3.32	1.00	3.32 (1.45; 5.00-1.00)
Condition	Privacy protection	4.00	3.42	2.25	3.42 (0.70; 5.00-2.25)
Condition	Usability	4.00	3.96	3.00	3.96 (0.55; 5.00-3.00)
Condition	Authority background	4.00	3.70	3.00	3.70 (0.49; 5.00-3.00)
Outcome	Willingness to use	4.00	3.74	1.33	3.74 (0.89; 5.00-1.33)

a For direct calibration, the observed maximum minus 1 was specified as the threshold for full membership, the observed mean as the crossover point, and the observed minimum as the threshold for full nonmembers hip.

#### Necessity Analysis

The results of the necessity analysis are presented in Table 4. The consistency values for all individual conditions and their negations were below 0.90; therefore, none met the prespecified criterion for a necessary condition for either high or low willingness to use. All six conditions were subsequently included in the configurational analysis to examine combinations associated with high and low willingness.

**Table 4.** The results of the necessity analysis.

Condition	High willingness to use (consistency)	High willingness to use (coverage)	Low willingness to use (consistency)	Low willingness to use (coverage)
Reward mechanism	0.74	0.62	0.58	0.62
~Reward mechanism	0.55	0.50	0.65	0.76
Interactivity	0.57	0.68	0.48	0.73
~Interactivity	0.77	0.54	0.79	0.70
Fun factor	0.81	0.73	0.46	0.53
~Fun factor	0.48	0.41	0.77	0.84
Privacy protection	0.84	0.68	0.64	0.65
~Privacy protection	0.57	0.55	0.68	0.85
Usability	0.48	0.55	0.56	0.81
~Usability	0.83	0.60	0.69	0.63
Authority background	0.37	0.49	0.45	0.77
~Authority background	0.83	0.54	0.70	0.59

#### Configurational Analysis

The standard analysis generated complex, parsimonious, and intermediate solutions for both high and low willingness to use gamified mHealth apps. The complete results for all 3 solution types are provided in Tables S1-S6 in Multimedia Appendix 4*.* The intermediate solutions were used as the primary basis for interpretation and are presented in Table 5.

**Table 5.** Intermediate solutions associated with high and low willingness to use gamified mobile health (mHealth) apps among preadmission patients with pituitary adenoma^a^.

Condition variables	High willingness to use^b^				Low willingness to use^c^				
	H1	H2	H3	H4	L1	L2	L3	L4	L5
Reward mechanism	●	⊗	⊗	^⊗^	^⊗^	⊗	^⊗^	•	●
Interactivity	•	●	^⊗^	^⊗^	^⊗^	^⊗^	•	•	•
Fun factor	•	•	•	^⊗^	⊗	⊗	⊗	⊗	•
Privacy protection	⊗	•	●	•	⊗		⊗	⊗	•
Usability	^⊗^	⊗	●	•		⊗	●		●
Authority background	⊗	^⊗^	^⊗^	•	^⊗^	•		•	⊗
Raw coverage	0.30	0.24	0.23	0.23	0.37	0.28	0.23	0.22	0.17
Unique coverage	0.11	0.04	0.07	0.09	0.16	0.10	0.03	0.08	0.04
Consistency	0.97	1.00	0.96	0.99	0.99	0.99	0.99	0.99	1.00

a A large solid circle indicates the presence of a core condition, and a small solid circle indicates the presence of a peripheral condition. A large crossed circle indicates the absence of a core condition, and a small crossed circle indicates the absence of a peripheral condition. A blank cell indicates that the condition may be either present or absent. Raw coverage indicates the proportion of outcome membership covered by a configuration, whereas unique coverage indicates the proportion covered exclusively by that configuration. The intermediate solutions were used as the primary basis for interpretation.

b Overall solution coverage: 0.51; overall solution consistency: 0.96.

c Overall solution coverage: 0.65; overall solution consistency: 0.99.

Four configurations were associated with high willingness to use: reward-driven engagement (H1), interactive enjoyment with privacy protection (H2), privacy and usability-supported enjoyment (H3), and authority-supported functional assurance (H4). The consistency values of the 4 configurations ranged from 0.960 to 0.997. The overall solution consistency was 0.964, and the overall solution coverage was 0.510. H1 had the highest raw coverage (0.298) and unique coverage (0.109).

Five configurations were associated with low willingness to use: broad feature deficit (L1), authority with engagement and usability deficits (L2), enjoyment and privacy deficit (L3), enjoyment and privacy deficit despite reward and authority support (L4), and authority-background deficit (L5). The consistency values of the five configurations ranged from 0.994 to 1.000. The overall solution consistency was 0.993, and the overall solution coverage was 0.645. L1 had the highest raw coverage (0.369) and unique coverage (0.157), whereas L5 had the lowest raw coverage (0.166).

## Discussion

### Principal Findings

No individual condition met the prespecified necessity threshold for high or low willingness to use gamified mHealth apps. Instead, multiple configurations of app-related conditions were associated with each outcome. These findings indicate that willingness was associated with different combinations of app-related conditions within the preadmission context and may not be adequately represented by examining individual conditions in isolation. The fsQCA identified several empirically observed configurations associated with high and low willingness. However, these configurations represent cross-sectional set relationships and should not be interpreted as mechanisms that produce willingness. These findings provide an exploratory configurational perspective on willingness to use gamified mHealth apps.

The SOR framework guided the interview questions and the organization of the qualitative findings. Zhan et al [33] similarly used the SOR framework to organize relationships among external stimuli, psychological responses, and behavioral intentions. The qualitative phase identified 6 app-related conditions relevant to willingness: reward mechanisms, interactivity, enjoyment, privacy protection, usability, and authority background. These 6 conditions were subsequently included in the fsQCA to examine their combinations in relation to willingness to use. Previous studies have reported associations between interactive or rewarding features and users’ engagement with digital interventions [34,35]. Privacy protection and usability have also been identified as relevant considerations in patients’ evaluations of mHealth technologies [36,37].

Reward-driven engagement (H1) configuration was characterized by the core presence of reward mechanisms and the core absence of privacy protection. Interactivity and enjoyment were present as peripheral conditions, whereas usability and authority background were absent as peripheral conditions. H1 had the highest raw coverage (0.298) and unique coverage (0.109) among the 4 high-willingness configurations. Thus, within the current sample, high willingness co-occurred with a combination emphasizing reward, interaction, and enjoyment, even when privacy protection, usability, and authority background were absent. Previous studies have also reported associations between rewarding or interactive features and engagement with digital interventions [34,35]. However, the present cross-sectional findings do not demonstrate that these features increased willingness or operated through social identification or positive affect. H2 was characterized by the core presence of interactivity and the core absence of reward mechanisms and usability. Enjoyment and privacy protection were present as peripheral conditions, whereas authority background was absent as a peripheral condition. This configuration indicates that high willingness co-occurred with interactivity, enjoyment, and privacy protection despite the absence of reward mechanisms, usability, and authority background. Previous studies have identified interactivity, enjoyment, and privacy protection as relevant considerations in users’ evaluations of digital health apps [36-38]. However, the present analysis did not test whether positive affect or trust mediated these associations.

H3 was characterized by the core presence of enjoyment, privacy protection, and usability and the core absence of reward mechanisms. Interactivity and authority background were absent as peripheral conditions. This configuration shows that high willingness was observed when enjoyment was combined with privacy protection and usability, even without reward mechanisms, interactivity, or authority background. Previous research has identified usability and privacy protection as relevant to patients’ evaluations of mHealth apps [36,37,39]. The current findings describe an observed combination of conditions and do not demonstrate that these features reduced technological barriers or increased adoption. H4 was characterized by the core presence of usability and authority background and the core absence of enjoyment. Privacy protection was present as a peripheral condition, whereas reward mechanisms and interactivity were absent as peripheral conditions. This configuration indicates that high willingness co-occurred with authority background, usability, and privacy protection in the absence of reward mechanisms, interactivity, and enjoyment. Previous studies have reported that professional endorsement, usability, privacy, and data security are relevant to patients’ evaluations of mHealth technologies [39,40]. Nevertheless, this configuration represents a cross-sectional association and does not demonstrate that authority background or functional assurance caused willingness or adoption.

To examine asymmetry between the 2 outcomes, we analyzed the configurations associated with low willingness to use gamified mHealth apps and identified 5 empirically observed patterns. Broad feature deficit (L1) was characterized by the core absence of enjoyment and privacy protection and the peripheral absence of reward mechanisms, interactivity, and authority background; usability was not decisive in this configuration. This pattern describes the co-occurrence of broad feature absence and low willingness but does not establish that these absences made the app unattractive or untrustworthy. L2 combined the core presence of authority background with the core absence of enjoyment and usability. Reward mechanisms and interactivity were absent as peripheral conditions, whereas privacy protection was not decisive. Thus, authority background co-occurred with low willingness when several engagement- and usability-related conditions were absent. The cross-sectional data do not establish rejection of use or long-term disengagement. L3 was characterized by the core presence of usability and the core absence of enjoyment. Interactivity was present as a peripheral condition, whereas reward mechanisms and privacy protection were absent as peripheral conditions; authority background was not decisive. This pattern indicates that interactivity and usability co-occurred with low willingness when enjoyment, reward mechanisms, and privacy protection were absent. Emotional responses and trust were not directly tested in this configuration [38-41]. L4 combined the core presence of reward mechanisms, interactivity, and authority background with the core absence of enjoyment and privacy protection; usability was not decisive. This configuration indicates that reward mechanisms, interactivity, and authority background did not invariably correspond to high willingness when enjoyment and privacy protection were absent. L5 was characterized by the core presence of reward mechanisms and usability and the core absence of authority background. Interactivity, enjoyment, and privacy protection were present as peripheral conditions. Thus, the absence of authority background co-occurred with low willingness even when the other 5 conditions were present [40]. Collectively, the 5 low-willingness configurations indicate that low willingness was associated with both broad feature absence and more specific combinations of present and absent conditions.

The configurations associated with high and low willingness were asymmetric rather than direct opposites. The same condition could be present in configurations associated with different outcomes, depending on the conditions with which it was combined. For example, reward mechanisms and interactivity were present in H1 but also in L4 and L5. Authority background was present in H4 but also in L2 and L4. Conversely, authority background was absent in H1-H3 and L5. These findings indicate that no single condition had a uniform relationship with willingness across all configurations.

### Limitations

This study has several limitations. First, participants were recruited from a single medical institution, and both the qualitative and quantitative samples were relatively small. Although the final 2 interviews generated no new codes or categories, indicating code saturation for the focused research question, the 10 interviews may not have achieved meaning saturation or captured the full heterogeneity of patients with PA. The quantitative sample of 120 participants may also limit the stability and generalizability of the identified configurations. Larger and more diverse samples from multiple institutions and geographic regions are therefore needed. Second, the eligibility criteria required participants to own and be able to use a smartphone and to have used an mHealth app within the previous month. These criteria may have favored patients with greater digital experience and at least some prior receptivity to mHealth. Therefore, the low-willingness configurations identified in this study represent relatively low willingness among patients with recent mHealth experience and should not be interpreted as representing individuals who categorically reject mHealth technologies. Future studies should include patients with a wider range of digital experience and initial attitudes toward mHealth. Third, the cross-sectional design assessed willingness at a single time point and could not establish causal relationships. Participants evaluated a description and slide presentation of a hypothetical gamified mHealth app rather than using a functioning app. Therefore, stated willingness should not be interpreted as actual adoption, sustained use, adherence, or clinical effectiveness. The identified configurations may inform preliminary app design but require validation through longitudinal studies using deployed apps and objective measures, such as app log data. Finally, although gamified design enhanced intention in some pathways, potential risks and boundaries should be considered. Excessive or poorly designed gamification may increase cognitive load and lead to engagement fatigue, particularly in high-stress contexts [42]. When gamification is perceived as distracting rather than supportive, its effect may weaken or even become counterproductive [43]. Thus, gamification may not be suitable for all patients or clinical settings, and its effectiveness likely depends on the fit among patient characteristics, disease stage, and clinical context.

### Innovation and Implications

The innovation of this study lies in examining gamified mHealth acceptance within the specific and time-limited preadmission pathway of patients with PA. Unlike studies that assess isolated predictors of general mHealth acceptance, this study linked patients’ qualitative accounts with SOR-guided instrument development and fsQCA to identify alternative feature configurations associated with both high and low willingness. This approach contributes disease- and stage-specific configurational evidence rather than assuming that findings from general, chronic disease, or postoperative settings are directly applicable to pre-admission care for PA. The configurations may inform the development of modular prototypes aligned with common preadmission tasks. Under H1, points, progress indicators, and interactive checklists could support the completion of admission documents, required examinations, and preoperative preparation tasks. Under H2, interactive disease education and question-and-answer activities could be combined with clear consent procedures and user-controlled data sharing. Under H3, simple navigation, minimal operation steps, and accessible privacy settings could facilitate access to examination schedules, hospitalization instructions, and symptom-reporting functions. Under H4, hospital identification, clinician-reviewed content, update dates, and transparent data-security information could be displayed prominently to clarify the professional source of PA-specific guidance.

The low-willingness configurations indicate that isolated features may not be sufficient: institutional authority without usable and engaging functions, interactivity without privacy protection, or multiple attractive features without clear professional provenance may still coincide with low willingness. Accordingly, developers could provide alternative feature modules rather than a uniform design and evaluate which combinations patients select and use during the preadmission period. These implications remain preliminary and require prospective testing with functioning apps, app-log data, and objective completion rates for preadmission tasks.

### Conclusions

Guided by the SOR framework, this exploratory sequential mixed methods study identified 6 app-related conditions relevant to the willingness of preadmission patients with PA to use gamified mHealth apps: reward mechanisms, interactivity, enjoyment, privacy protection, usability, and authority background. The fsQCA identified 4 configurations associated with high willingness—reward-driven engagement, interactive enjoyment with privacy protection, privacy and usability-supported enjoyment, and authority-supported functional assurance—and 5 configurations associated with low willingness. These findings provide a disease- and stage-specific configurational perspective by showing that willingness was associated with alternative feature combinations rather than any single app feature. The configurations may inform the development of modular preadmission app prototypes incorporating task rewards and progress feedback, interactive education with privacy controls, simplified access to examination and hospitalization instructions, and hospital-endorsed, clinician-reviewed information. However, the findings represent exploratory associations with willingness toward a hypothetical app and do not establish causal effects, actual adoption, or sustained use. Prospective evaluation using functioning apps, larger multicenter samples, and objective app-use data is required before clinical implementation.

## Supplementary material

10.2196/92924 Multimedia Appendix 1 Interview guide on factors influencing the willingness to use gamified mobile health (mHealth) apps outline.

10.2196/92924 Multimedia Appendix 2 Examples of features in gamified health mHealth apps.

10.2196/92924 Multimedia Appendix 3 Survey on factors influencing the willingness to use gamified mHealth apps in patients with pituitary adenoma during preadmission.

10.2196/92924 Multimedia Appendix 4 Complex, parsimonious, and intermediate fuzzy-set qualitative comparative analysis solutions for high and low willingness to use gamified mHealth apps among pre-admission patients with pituitary adenoma.

## Abbreviations

EHR: electronic health record
fsQCA: fuzzy-set qualitative comparative analysis
KMO: Kaiser-Meyer-Olkin
mHealth: mobile health
PA: pituitary adenoma
QCA: qualitative comparative analysis
SOR: stimulus-organism-response
