# Supplementary material for: Willingness to Use Gamified mHealth Apps Among Preadmission Patients With Pituitary Adenoma: Exploratory Sequential Mixed Methods Study
Source: JMIR Serious Games. 2026 Nov 9;14:e92924. doi: 10.2196/92924 (PMC13747270; doi:10.2196/92924)
Supplement: Supplementary file 1 [file games-v14-e92924-s001.docx]

**Interview guide on factors influencing the willingness to use gamified mobile health (mHealth) apps outline**

1. **Introduction by the Interviewer**
   Good morning/afternoon/evening, and thank you for taking your valuable time to join our interview via Tencent Meeting. The topic of our discussion today is the factors influencing the willingness of patients with pituitary adenoma in the pre-admission stage to use Gamified mHealth Apps. I am a nurse in the Department of Neurosurgery. You have been invited today because we would like to hear the views of patients like yourself on this topic. The results of this research will help us better understand your perspectives and apply them to our future studies.
2. **Principles of Semi-structured Interviews**
   - There are no right or wrong answers to the patients’ responses.
   - Minimize interference during the interview process, allowing the patient to lead the conversation.
   - Ensure that core questions are covered, while also allowing the respondent to provide additional information.
   - It is recommended that the patient use a camera; however, if the patient declines, their preferences will be respected.
   - Respect the boundaries of the respondent and avoid pressuring them.
   - During the interview, if the patient experiences discomfort, the interview can be paused and rescheduled for another time.
3. **Filling in Personal Information**
   Before we begin with the first question, please assist us by filling out your personal information. You will need to answer the following questions:
   - Gender
   - Age
   - Marital Status
   - Occupation
   - Education Level
   - Type of Disease
   - Accompanying Symptoms
   - Do you own a smartphone?
   - Do you use your smartphone to send messages or browse the internet at least once a day?
   - Do you use health apps to understand your illness?
   - Are you interested in Gamified mobile healthcare programs?
   - Are you familiar with mobile phone games?
4. **Questions**
   1. **About Gamified mHealth Apps**
      Have you heard of or used any Gamified mHealth Apps before? Considering the challenges you face during the pre-admission stage, what are your thoughts on using a Gamified mHealth App for the full disease management of pituitary adenoma in the pre-admission stage?
   2. **Stimuli Factors of Gamified mHealth Apps**
      1. What factors would encourage you to choose and use this Gamified mHealth App? Please be specific.
      2. What factors would prevent you from choosing to use this app?
      3. What type of gamification would you prefer? For example: storyline tasks, point exchange, or virtual character development?
   3. **Organism Responses**
      1. Please explain why these factors would encourage you to use the app. What specific experience did it provide you?
      2. Please explain why these factors would prevent you from using the app. What specific experience did it provide you?
   4. **Organism Behavior**
      1. Would you recommend this app to other patients with pituitary adenoma? Why?
      2. If you stopped using this app, what might be the possible reasons?
   5. **PPT Presentation**
      To deepen your impression, we have prepared a presentation of the functions currently available in Gamified mHealth Apps in the software store. Please share your thoughts based on these features and tell us which features you think are necessary, which are unnecessary, and which should be added.

**6. Ending**
Thank you very much for participating in our interview and sharing this valuable information.

- Would you be willing to review and verify the accuracy of the interview content after it is transcribed?
- Would you be interested in receiving a summary of the study results once the research is completed?
