# Supplementary material for: Willingness to Use Gamified mHealth Apps Among Preadmission Patients With Pituitary Adenoma: Exploratory Sequential Mixed Methods Study
Source: JMIR Serious Games. 2026 Nov 9;14:e92924. doi: 10.2196/92924 (PMC13747270; doi:10.2196/92924)
Supplement: Supplementary file 2 [file games-v14-e92924-s003.docx]

**Survey on factors influencing the willingness to use gamified mHealth apps in patients with pituitary adenoma during pre-admission.**

**Instructions**:
This questionnaire aims to understand the factors influencing patients' willingness to use Gamified mHealth Apps in order to improve the relevant designs. Please answer based on your actual situation. All data will be used for research analysis and kept strictly confidential.

**I. Basic Information**

1. **Your age**
   □ 18-44
   □ 45-59
   □ 60-74
   □ 75 and above
2. **Your gender**
   □ Male
   □ Female
3. **Your education level**
   □ Primary school
   □ Middle school
   □ High school or vocational school
   □ Bachelor's degree
   □ Master's degree
4. **Your employment status**
   □ Full-time
   □ Part-time
   □ Student
   □ Unemployed
   □ Retired
   □ Other
5. **Type of pituitary adenoma**
   □ Functional (e.g., prolactinoma, growth hormone tumor, ACTH tumor, TSH tumor, gonadotropin tumor)
   □ Non-functional
   □ Uncertain
6. **Tumor size**
   □ Microadenoma (<1 cm)
   □ Macroadenoma (>1 cm)
7. **How do you usually obtain health information?** (Multiple answers allowed)
   □ Doctors or healthcare professionals
   □ Family or friends
   □ Fellow patients
   □ Online search or medical apps
   □ Other (please specify): ___________
8. **Current main symptoms**
   □ Visual impairment
   □ Headache
   □ Hormonal abnormalities (e.g., menstrual irregularities, acromegaly)
   □ Fatigue
   □ Other

**II. Screening Checklist for Factors Influencing the Willingness to Use Gamified mHealth Apps in Patients with PA during Pre-admission**

**A. Reward Mechanism**

1. I believe that point rewards (e.g., earning points for completing tasks to redeem gifts) motivate me to continue using the program.
   □ Strongly agree □ Agree □ Neutral □ Disagree □ Strongly disagree
2. Virtual achievements (e.g., badges, level-ups) make me more willing to complete health tasks (e.g., watching health education videos).
   □ Strongly agree □ Agree □ Neutral □ Disagree □ Strongly disagree
3. I would like to unlock exclusive health knowledge or useful tools (e.g., symptom self-assessment guides, personalized exercise plans) through the program's point rewards.
   □ Strongly agree □ Agree □ Neutral □ Disagree □ Strongly disagree
4. Progress bars or achievement indicators (e.g., "80% of the treatment completed") enhance my confidence in treatment.
   □ Strongly agree □ Agree □ Neutral □ Disagree □ Strongly disagree

**B. Interactivity**

1. Having the ability to interact with other patients (e.g., sharing experiences, mutual encouragement) would increase my willingness to use the app.
   □ Strongly agree □ Agree □ Neutral □ Disagree □ Strongly disagree
2. I would like to consult with my attending physician online or provide feedback about my condition through the app.
   □ Strongly agree □ Agree □ Neutral □ Disagree □ Strongly disagree
3. Real-time feedback (e.g., synchronized test data, symptom improvement suggestions) is important to me.
   □ Strongly agree □ Agree □ Neutral □ Disagree □ Strongly disagree
4. I do not need to interact with other patients or doctors and prefer to use only the personal functions of the program. (Reverse score)
   □ Strongly agree □ Agree □ Neutral □ Disagree □ Strongly disagree

**C. Fun Factor**

1. Storylines or role-playing features in the program (e.g., “health adventure games”) make me more interested in participating.
   □ Strongly agree □ Agree □ Neutral □ Disagree □ Strongly disagree
2. A variety of task formats (e.g., quizzes, mini-games) is more appealing to me than a single task format.
   □ Strongly agree □ Agree □ Neutral □ Disagree □ Strongly disagree
3. Dynamic visual designs (e.g., animations, color changes) enhance my user experience.
   □ Strongly agree □ Agree □ Neutral □ Disagree □ Strongly disagree
4. I do not require fun elements in the program, as long as the functions are practical. (Reverse score)
   □ Strongly agree □ Agree □ Neutral □ Disagree □ Strongly disagree

**D. Privacy Protection**

1. I am concerned that the app may disclose my health data (e.g., medical records, medication history).
   □ Strongly agree □ Agree □ Neutral □ Disagree □ Strongly disagree
2. The program should clearly inform me of the scope of data usage and obtain my authorization.
   □ Strongly agree □ Agree □ Neutral □ Disagree □ Strongly disagree
3. I am willing to anonymously share treatment data to help medical research.
   □ Strongly agree □ Agree □ Neutral □ Disagree □ Strongly disagree
4. The app providing a data deletion function (e.g., one-click history deletion) would increase my trust in the app.
   □ Strongly agree □ Agree □ Neutral □ Disagree □ Strongly disagree

**E. Usability of the App**

1. The app interface is clear, and I can quickly find the functions I need (e.g., task tracking, goal achievement).
   □ Strongly agree □ Agree □ Neutral □ Disagree □ Strongly disagree
2. The process is simple, and I can use the app independently without guidance.
   □ Strongly agree □ Agree □ Neutral □ Disagree □ Strongly disagree
3. The voice command feature (e.g., voice input for symptoms) reduces the difficulty of using the app.
   □ Strongly agree □ Agree □ Neutral □ Disagree □ Strongly disagree
4. Even if the app is complex to use, I would patiently learn how to use it. (Reverse score)
   □ Strongly agree □ Agree □ Neutral □ Disagree □ Strongly disagree

**F. Authority Background Support**

1. Whether the program is recommended by a hospital does not affect my willingness to use it. (Reverse score)
   □ Strongly agree □ Agree □ Neutral □ Disagree □ Strongly disagree
2. If the program’s design (e.g., health tasks, feedback mechanisms) is developed with the involvement of my attending physician team, I would be more willing to use it.
   □ Strongly agree □ Agree □ Neutral □ Disagree □ Strongly disagree
3. A medical program with an authoritative background (e.g., supported by well-known hospitals or physician teams) would increase my trust in the app.
   □ Strongly agree □ Agree □ Neutral □ Disagree □ Strongly disagree
4. I hope the health advice in the app is based on guidelines from authoritative organizations (e.g., pituitary adenoma treatment standards).
   □ Strongly agree □ Agree □ Neutral □ Disagree □ Strongly disagree

**G. Willingness to Use Gamified Mobile Programs**

1. If the program includes the above features, the likelihood of my continued use during hospitalization is:
   □ Very likely □ Likely □ Neutral □ Unlikely □ Very unlikely
2. I would recommend this program to other patients with pituitary adenoma.
   □ Strongly agree □ Agree □ Neutral □ Disagree □ Strongly disagree
3. I think this program is more suitable for my needs than traditional health management tools (e.g., paper records, general medical apps).
   □ Strongly agree □ Agree □ Neutral □ Disagree □ Strongly disagree
