# Supplementary material for: Willingness to Use Gamified mHealth Apps Among Preadmission Patients With Pituitary Adenoma: Exploratory Sequential Mixed Methods Study
Source: JMIR Serious Games. 2026 Nov 9;14:e92924. doi: 10.2196/92924 (PMC13747270; doi:10.2196/92924)
Supplement: Supplementary file 3 [file games-v14-e92924-s004.docx]

**Complex, parsimonious, and intermediate fuzzy-set qualitative comparative analysis solutions for high and low willingness to use gamified mHealth apps among pre-admission patients with pituitary adenoma.**

**Table S1. Complex solution associated with high willingness to use**

| **Condition or fit statistic** | **CH1** | **CH2** | **CH3** | **CH4** |
| --- | --- | --- | --- | --- |
| Reward Mechanism | ● | ⊗ | ⊗ | ⊗ |
| Interactivity | ● | ● | ⊗ | ⊗ |
| Fun Factor | ● | ● | ● | ⊗ |
| Privacy Protection | ⊗ | ● | ● | ● |
| Usability | ⊗ | ⊗ | ● | ● |
| Authority Background | ⊗ | ⊗ | ⊗ | ● |
| Raw coverage | 0.298 | 0.237 | 0.225 | 0.232 |
| Unique coverage | 0.109 | 0.042 | 0.067 | 0.089 |
| Consistency | 0.971 | 0.997 | 0.960 | 0.990 |
| **Overall solution coverage** | **0.510** | | | |
| **Overall solution consistency** | **0.964** | | | |

**Note.** ● condition present; ⊗ condition absent. A blank cell indicates that the condition is not included in the solution term.

**Table S2. Parsimonious solution associated with high willingness to use**

| **Condition or fit statistic** | **PH1** | **PH2** | **PH3** |
| --- | --- | --- | --- |
| Reward Mechanism | ⊗ | ● | ⊗ |
| Interactivity |  |  | ● |
| Fun Factor |  |  |  |
| Privacy Protection | ● | ⊗ |  |
| Usability | ● |  | ⊗ |
| Authority Background |  | ⊗ |  |
| Raw coverage | 0.336 | 0.404 | 0.279 |
| Unique coverage | 0.123 | 0.177 | 0.048 |
| Consistency | 0.887 | 0.851 | 0.885 |
| **Overall solution coverage** | **0.608** | | |
| **Overall solution consistency** | **0.814** | | |

**Note.** ● condition present; ⊗ condition absent. A blank cell indicates that the condition is not included in the solution term.

**Table S3. Intermediate solution associated with high willingness to use**

| **Condition or fit statistic** | **IH1** | **IH2** | **IH3** | **IH4** |
| --- | --- | --- | --- | --- |
| Reward Mechanism | ● | ⊗ | ⊗ | ⊗ |
| Interactivity | ● | ● | ⊗ | ⊗ |
| Fun Factor | ● | ● | ● | ⊗ |
| Privacy Protection | ⊗ | ● | ● | ● |
| Usability | ⊗ | ⊗ | ● | ● |
| Authority Background | ⊗ | ⊗ | ⊗ | ● |
| Raw coverage | 0.298 | 0.237 | 0.225 | 0.232 |
| Unique coverage | 0.109 | 0.042 | 0.067 | 0.089 |
| Consistency | 0.971 | 0.997 | 0.960 | 0.990 |
| **Overall solution coverage** | **0.510** | | | |
| **Overall solution consistency** | **0.964** | | | |

**Note.** ● core condition present; ⊗ core condition absent; ● peripheral condition present; ⊗ peripheral condition absent. A blank cell indicates that the condition may be either present or absent. Core and peripheral conditions were identified by comparing the parsimonious and intermediate solutions.

**Table S4. Complex solution associated with low willingness to use**

| **Condition or fit statistic** | **CL1** | **CL2** | **CL3** | **CL4** | **CL5** |
| --- | --- | --- | --- | --- | --- |
| Reward Mechanism | ⊗ | ⊗ | ⊗ | ● | ● |
| Interactivity | ⊗ | ⊗ | ● | ● | ● |
| Fun Factor | ⊗ | ⊗ | ⊗ | ⊗ | ● |
| Privacy Protection | ⊗ |  | ⊗ | ⊗ | ● |
| Usability |  | ⊗ | ● |  | ● |
| Authority Background | ⊗ | ● |  | ● | ⊗ |
| Raw coverage | 0.369 | 0.276 | 0.227 | 0.215 | 0.166 |
| Unique coverage | 0.157 | 0.098 | 0.032 | 0.080 | 0.045 |
| Consistency | 0.995 | 0.994 | 0.995 | 0.994 | 1.000 |
| **Overall solution coverage** | **0.645** | | | | |
| **Overall solution consistency** | **0.993** | | | | |

**Note.** ● condition present; ⊗ condition absent. A blank cell indicates that the condition is not included in the solution term.

**Table S5. Parsimonious solution associated with low willingness to use**

| **Condition or fit statistic** | **PL1** | **PL2** | **PL3** |
| --- | --- | --- | --- |
| Reward Mechanism |  | ⊗ | ● |
| Interactivity |  |  |  |
| Fun Factor | ⊗ | ⊗ |  |
| Privacy Protection | ⊗ |  |  |
| Usability |  | ⊗ | ● |
| Authority Background |  |  | ⊗ |
| Raw coverage | 0.605 | 0.425 | 0.242 |
| Unique coverage | 0.238 | 0.089 | 0.076 |
| Consistency | 0.968 | 0.918 | 0.922 |
| **Overall solution coverage** | **0.773** | | |
| **Overall solution consistency** | **0.920** | | |

**Note.** ● condition present; ⊗ condition absent. A blank cell indicates that the condition is not included in the solution term.

**Table S6. Intermediate solution associated with low willingness to use**

| **Condition or fit statistic** | **IL1** | **IL2** | **IL3** | **IL4** | **IL5** |
| --- | --- | --- | --- | --- | --- |
| Reward Mechanism | ⊗ | ⊗ | ⊗ | ● | ● |
| Interactivity | ⊗ | ⊗ | ● | ● | ● |
| Fun Factor | ⊗ | ⊗ | ⊗ | ⊗ | ● |
| Privacy Protection | ⊗ |  | ⊗ | ⊗ | ● |
| Usability |  | ⊗ | ● |  | ● |
| Authority Background | ⊗ | ● |  | ● | ⊗ |
| Raw coverage | 0.369 | 0.276 | 0.227 | 0.215 | 0.166 |
| Unique coverage | 0.157 | 0.098 | 0.032 | 0.080 | 0.045 |
| Consistency | 0.995 | 0.994 | 0.995 | 0.994 | 1.000 |
| **Overall solution coverage** | **0.645** | | | | |
| **Overall solution consistency** | **0.993** | | | | |

**Note.** ● core condition present; ⊗ core condition absent; ● peripheral condition present; ⊗ peripheral condition absent. A blank cell indicates that the condition may be either present or absent. Core and peripheral conditions were identified by comparing the parsimonious and intermediate solutions.
